# Risk of Cardiovascular Diseases Associated with PCOS in India: A Review

**DOI:** 10.2174/011573403X349035250408050637

**Published:** 2025-04-23

**Authors:** Amandeep Kaur, Ranjeet Kumar, Hardik Kumar, Sonakshi Garg, Abhishek Vijukumar, Dharmendra Kumar

**Affiliations:** 1 Department of Pharmacy Practice, ISF College of Pharmacy, Moga, Punjab, India;; 2 Narayan Institute of Pharmacy, Gopal Narayan Singh University, Sasaram, Rohtas, Bihar, 821305, India

**Keywords:** Polycystic ovary syndrome, cardiovascular disease, hypertension, insulin resistance, diabetes mellitus, dyslipidemia

## Abstract

In the modern world, Polycystic Ovary Syndrome (PCOS) is thought to be the most prevalent endocrine condition affecting women. Compared to their normal counterparts, PCOS patients have higher rates of morbidity and death because they are more susceptible to these anomalies from an early age. Cardiovascular disease (CVD) and PCOS are prevalent in women. PCOS often results from a combination of hereditary and environmental causes. Insulin resistance (IR) is considered the primary cause of several metabolic risk factors, such as Type 2 Diabetes Mellitus (T2DM), Metabolic Syndrome (MetS), dyslipidemia, obesity, and hypertension (HTN). Additionally, patients with PCOS may also have elevated levels of non-traditional factors, including C-reactive protein (CRP), carotid intima-media thickness (IMT), coronary artery calcification (CAC), as well as endothelial dysfunction, which raises the likelihood of complications from CVD. This review utilizes statistics and data mostly sourced from research in India, offering insight into the nation's distinct PCOS prevalence and related cardiovascular risks. To lessen the impact of PCOS in the modern world, prompt identification and effective management of these warning signs with food, lifestyle changes, and/or medication are crucial. The research that examined the potential impact of PCOS on the most prevalent CVD-hypertension, insulin resistance, obesity, malignancy, diabetes mellitus, and dyslipidemia-is reviewed in this study. Measuring subclinical atherosclerosis, such as coronary artery calcium or carotid plaque, might help inform shared decision-making over the start of statin therapy when CVD risk is unknown.

## 1 INTRODUCTION

Polycystic Ovary Syndrome (PCOS) is a complex illness with a broad spectrum of symptoms that affects not only adolescent girls and women going through menopause but also females in their reproductive years [1]. Women are more likely to have cardiovascular disease (CVD) than males due to PCOS. Age is still one of the most significant risk factors for the onset of atherosclerosis and dying from a cardiovascular event, and CVD is still the leading cause of mortality in women [2]. Environmental factors, heredity, insulin resistance, eating patterns, an unhealthy lifestyle, utilizing personal care products with carcinogenic preservation agents, family history, inflammatory variables, altered steroidogenesis, obesity, and other factors are some of the causes of PCOS. According to a number of publications, the hormone-disturbing properties of triclosan, an antibacterial ingredient found in toothpaste, maybe the cause of PCOS [3]. Infertility and androgen excess symptoms, such as virilization, hirsutism, acne, alopecia, and irregular menstruation, such as amenorrhea and dysfunctional bleeding, are common in women with PCOS [4]. Numerous conventional CVD risk factors are highly prevalent in PCOS patients. These comprise modifiable risk factors, such as diabetes, obesity, hypertension, and dyslipidemia [5], as shown in Fig. (**1**). Given that this patient population can improve their metabolic profile and lower their risk of CVD by combining medication and lifestyle management, CVD risk screening is still essential [6]. For women with PCOS at greater risk of atherosclerotic cardiovascular disease, the initiation of statin therapy may be considered as an aspect of a complete risk management plan [7]. Measuring subclinical atherosclerosis, such as coronary artery calcium or carotid plaque, might help inform shared decision-making over the start of statin therapy when CVD risk is unknown. In insulin-resistant populations, other drugs like metformin and agonists of glucagon-like peptide-1 may also be helpful in lowering the risk of CVD [8]. Further investigation is required to determine the most effective strategies for mitigating the cardiovascular disease risk linked to PCOS [9].

## 2 EPIDEMIOLOGY

According to the few studies that have investigated the prevalence of PCOS in India, the bulk of the sampling that was employed was based on convenience, which may not correctly reflect the actual prevalence of PCOS in the community. A preliminary cross-sectional study that was conducted in Tamil Nadu to evaluate early adolescent females found that the prevalence of PCOS was 18% [10]. The findings indicated that urban women had a higher prevalence of PCOS compared to rural women. A similar community-based study conducted in Mumbai found that the overall prevalence of PCOS was 10.7% according to the Androgen Excessive Association criteria and 22.5% according to the Rotterdam criteria [11]. In the course of a research project involving medical graduates from a private college of medicine in south India, the modified Cronin survey, which consisted of ten questions, was used. A high prevalence of mental problems and PCOS was shown to be a widespread condition among the patients, according to the findings [12]. Women who were preparing to attend college and who suffered from hirsutism and irregular periods were the subjects of a research that was found to have been conducted in Lucknow and published. A range of ages, from 18 to 25, comprised the participants in the study. Using the standards that were set by the National Institutes of Health, it was found that the estimated prevalence of the individuals who participated was just 3.7% [13]. In another study, it was discovered that 9.13% of the young women who were residing in a residential institution in Andhra Pradesh met the Rotterdam criteria for PCOS [14, 15], and 4-10% of women who were of reproductive age had PCOS, according to research that was carried out on a global scale [16]. Consequently, between 3.7 and 22.5% of Indians have PCOS, based on the limited evidence that is currently available [17-19].

## 3 CARDIO-METABOLIC RISK FACTORS AND ASSOCIATED OUTCOMES OF PCOS

### 3.1 Insulin Resistance and Type 2 Diabetes Mellitus

Insulin resistance (IR) is the result of insulin's inability to consistently transport glucose into body cells [20]. Furthermore, deficient tyrosine phosphorylation of the insulin receptors and insulin receptor substrate-1 may result in metabolic disorders in muscle cells, adipocytes, and the ovaries. Generally, IR is caused by an interruption in the post-binding signaling of insulin due to a rise in serine phosphorylation [21, 22]. Some alternative mechanisms of IR that can increase the risk of T2DM include Reduced Glucose Transporter 4 (GLUT4) in subcutaneous adipocytes, impaired liver insulin clearance, abnormalities in mitochondrial function, as well as serine kinase stimulation within the Mitogen-Activated Protein Kinase/Extracellular Signal-Regulated Kinase (MAPK-ERK) Pathway. Furthermore, flawed tyrosine phosphorylation of the insulin receptors alongside insulin receptor substrate-1 may trigger metabolic disorders in muscle cells and adipocytes alongside the ovaries. Generally, IR is caused by an error within the post-binding signaling of insulin due to a rise in serine phosphorylation [23, 24].

Additional mechanisms contributing to IR that heighten the risk of developing T2DM encompass a reduction in GLUT4 in subcutaneous adipocytes, diminished insulin clearance in the liver due to mitochondrial dysfunction, and the activation of serine kinase within the MAPK-ERK pathway. Additional mechanisms contributing to IR that may elevate the risk of developing T2DM encompass a reduction in GLUT4 in subcutaneous adipocytes, diminished insulin clearance in the liver due to mitochondrial dysfunction, and the activation of serine kinase within the MAPK-ERK pathway [25]. Normal-weight girls with PCOS had a three-fold greater risk of T2DM incidence when compared to weight-matched healthy controls [26]. Ovalle *et al.* conducted a review of the literature and discovered that the likelihood of IR was elevated in fifty to eighty percent of women who had PCOS. In addition, a prospective controlled study that included 254 patients with PCOS found that patients with PCOS had higher prevalence rates of Impaired Glucose Tolerance (IGT) and type 2 diabetes mellitus, with rates of 31.3% and 7.5%, respectively. This was in contrast to the rates of 14% and 0%, respectively, in age- and weight-matched individuals who did not have PCOS [23]. IGT has also been linked to an increased risk of type 2 diabetes, cardiovascular disease, and mortality in the general population in recent years [27]. IGT and T2DM are more frequent in persons with PCOS -31.3% and 7.5%, respectively-compared to age- and weight-matched patients without PCOS [23]. IGT has additionally been recently linked to a higher likelihood of T2DM, CVD, as well as mortality in people in general [27].

### 3.2 Metabolic Syndrome

Hyperglycemia, characterised by fasting glucose levels of 5.6 mmol/L or greater, low HDL (below 1.29 mmol/L), elevated triglycerides (exceeding 1.7 mmol/L), central obesity (an increased waist circumference relative to the general population and the individual's country of residence), and heightened diastolic or systolic blood pressure (surpassing 130/80 mmHg) constitute the criteria for metabolic syndrome [28].

Insulin resistance is the underlying cause of metabolic syndrome in women with PCOS. Every clinical symptom associated with metabolic syndrome may be connected to an individual cause. The onset of hyperglycemia may result from compensatory hyperinsulinemia, perhaps involving the loss of pancreatic beta-cells due to insulin resistance [29, 30]. In the end, metabolic syndrome is the result of the accumulation of a number of metabolic characteristics that are known to be risk factors for cardiovascular illnesses. These characteristics include obesity, dyslipidaemia, type 2 diabetes mellitus, impaired glucose tolerance, insulin resistance, and hypertension [23].

### 3.3 Dyslipidemia

Dyslipidaemia, which is one of the most frequent metabolic abnormalities linked with PCOS, affects more than seventy percent of women who have the illness [31]. Patients with PCOS exhibited higher levels of LDL cholesterol and non-HDL cholesterol, even in situations when their Body Mass Index (BMI) was equivalent [32]. Among older women who have PCOS, the incidence of dyslipidaemia has only been described in a few studies. According to the findings of cross-sectional research, there was no significant difference in serum lipid levels between perimenopausal PCOS women and controls [33]. No statistically significant difference was observed in the likelihood of developing dyslipidaemia between women with PCOS and those who served as controls after the age of 40. On a similar note, the researchers of the retrospective cohort study discovered that the blood lipid levels of individuals with PCOS and controls, who had a mean age of 46.7 years, were comparable until the age of 45 to 50 years [34]. As a consequence of this, people who have PCOS may have dyslipidaemia at a higher frequency. People with PCOS have increased levels of LDL and non-HDL cholesterol, as well as recognised changes in the levels of both triglycerides and HDL cholesterol, regardless of their BMI. All individuals with PCOS should have their dyslipidaemia, particularly their levels of LDL and non-HDL cholesterol, evaluated in order to get the most effective cardiovascular risk prevention [35, 36].

### 3.4 Hypertension

Several studies have revealed that PCOS females have a greater incidence of hypertension [4, 37]. In order to define Hypertension (HTN), the American College of Cardiology (ACC) and the American Heart Association (AHA) employ the criteria of Systolic Blood Pressure (SBP) that is more than or equal to 130 mmHg and diastolic blood pressure that is greater than or equal to 80 mmHg. When these criteria were considered, it was shown that hypertension was 24% more prevalent in PCOS patients compared to healthy females [38, 39]. In PCOS individuals, renin-angiotensin system activation results in hypertension [40]. The results of a recent meta-analysis revealed that patients with PCOS had a greater prevalence of hypertension compared to the control group [41]. During this time period, it was determined that only women of reproductive age had this condition; menopausal women who previously had PCOS were not found to have this condition [42]. Those who experienced signs of PCOS and had an average age of 45 years had increased rates of both body mass index and hypertension when compared to those who had regular periods and were considered controlled [43, 44]. Significant elevations in both systolic and diastolic blood pressure were seen in PCOS groups collectively when compared to those who acted as controls [44]. Even while other studies did not identify any detectable differences in the prevalence of hypertension between the elderly female PCOS group and the general population, it is important to highlight that women who have PCOS are more likely to have hypertension [45]. Often, women with PCOS are more likely to have high blood pressure. Further study is now needed to regulate the risk of hypertension in older PCOS patients who have gone past menopause [46].

### 3.5 Obesity

One concomitant risk factor for PCOS individuals is obesity [47]. Research indicates that IR is the primary cause of obesity because it results in hyperinsulinemia, which triggers the ovary and adipose tissue to synthesize steroids. This, in turn, lowers hepatic production for sex hormone-binding globulin, increasing free androgens. If elevated over an extended period, this can lead to central obesity through the accumulation of visceral fat, aggravating the symptoms of PCOS, and so on [48, 49].

Compared to healthy controls, women with PCOS who were over 45 had greater average BMI values and wider waist circumferences [50]. Three distinct periods of the reproductive life were represented in the study by women, ages 18 to 34, 35 to 40, and 41 to 55, as well as healthy controls. PCOS patients had higher BMI as well as waist-to-hip ratio requirements during the initial and late phases of pregnancy than controls. However, there was no distinction in the groups during the perimenopausal phase [51].

Consequently, obese individuals with PCOS are more likely than their leaner counterparts to have worse reproductive and overall medical outcomes; as a result, they are more likely to receive diagnosis and therapy earlier, which may account for the overestimated link between weight gain and PCOS [52].

### 3.6 Cancer

Female PCOS patients have associated risks for endometrial cancer, such as obesity, prolonged unopposed estrogen, and nulliparity. The likelihood of endometrial cancer was shown to be significantly greater in females with PCOS, but there was no appreciable change in the risk of breast or ovarian cancer [53]. After studies with participants older than 54 years were removed from the data, the risk of endometrial and ovarian cancers rose significantly for females with PCOS, but no significant risk was detected for cancer of the breast [54].

Using data gathered from the Danish National Patients Register, the author calculated an overall 4-fold increased risk of Endometrial Cancer (EC); however, no connection between PCOS and either ovarian or breast cancer was found. In conclusion, women experiencing PCOS and healthcare professionals should be aware of a 2 to 6-fold increased risk of endometrial cancer, even if the small records of occurrences implicated restrict this research [55].

## 4 FUTURE PERSPECTIVE

One can lower the likelihood of having T2DM and its consequences, including PCOS, by adopting a better lifestyle. Novel pharmacological methods for the ongoing treatment and cure of PCOS may result from an understanding of the connection between T2DM and its condition. Natural supplements like β-glucan, which may have therapeutic advantages, can lower the risk of diabetes. Moreover, finding practical solutions is hampered by our limited understanding of the intricate relationships among β-glucan structures along with effects, methods of extraction, and clinical trials.

## 5 LIMITATIONS

This article solely considers cardio-metabolic components of PCOS, omitting additional risk factors that may be considered independently, such as cigarette smoking, menopause, anxiety, family history, and a multifactorial cause of PCOS that involves both hereditary and environmental variables.

## CONCLUSION

During the reproductive phase, women with PCOS are more likely to acquire significant CVD risk factors, such as obesity, dyslipidemia, diabetes, impaired glucose tolerance, and metabolic syndrome. On the other hand, nothing is known about the long-term impact of PCOS on health beyond menopause. This article has explored the complex interaction that PCOS has with our body functions by highlighting a number of cardiovascular and metabolic disorders, including endothelial dysfunction, T2DM, IGT, atherosclerosis, and hypertension. It has also explained the pathophysiology of each disorder.

Based on the aforementioned research, women with PCOS have a much higher lifetime risk of having cardio-metabolic illnesses than women without PCOS. However, if these abnormalities are detected early on, they can be treated or avoided to prevent worsening of the condition. This suggests that to combat this disease of the modern period, a large number of research and extensive screening programs are required. A few studies, nonetheless, have not shown any connection at all between PCOS and cardio-metabolic outcomes.

## Figures and Tables

**Fig (1):**
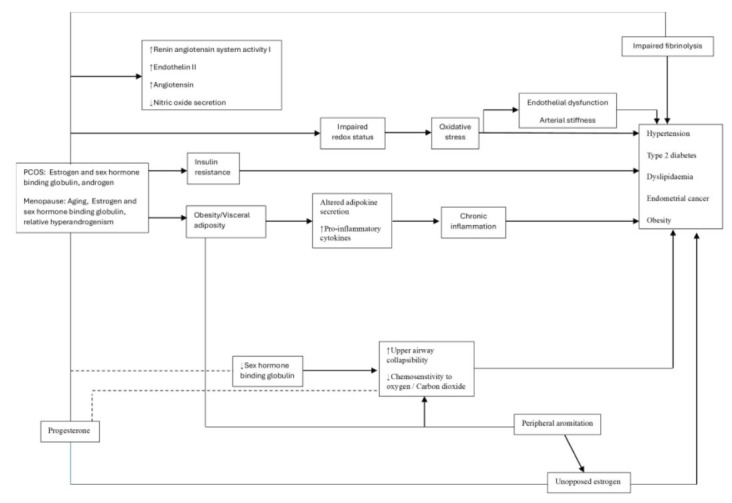
Menopause and PCOS schematic diagram: Pathological pathways with similar long-term health effects.

## LIST OF ABBREVIATIONS

CAC: Coronary Artery Calcification
CRP: C-reactive Protein
CVD: Cardiovascular Disease
HTN: Hypertension
IMT: Carotid Intima-media Thickness
IR: Insulin Resistance
MetS: Metabolic Syndrome
T2DM: Type 2 Diabetes Mellitus
